# High-Throughput Characterization of Blood Serum Proteomics of IBD Patients with Respect to Aging and Genetic Factors

**DOI:** 10.1371/journal.pgen.1006565

**Published:** 2017-01-27

**Authors:** Antonio F. Di Narzo, Shannon E. Telesco, Carrie Brodmerkel, Carmen Argmann, Lauren A. Peters, Katherine Li, Brian Kidd, Joel Dudley, Judy Cho, Eric E. Schadt, Andrew Kasarskis, Radu Dobrin, Ke Hao

**Affiliations:** 1 Department of Genetics and Genomic Sciences, Icahn School of Medicine at Mount Sinai, New York, New York, United States of America; 2 Icahn Institute of Genomics and Multiscale Biology, Icahn School of Medicine at Mount Sinai, New York, New York, United States of America; 3 Janssen R&D, LLC, Spring House, Pennsylvania, United States of America; 4 Graduate School of Biomedical Sciences, Icahn School of Medicine at Mount Sinai, New York, New York, United States of America; Stanford University School of Medicine, UNITED STATES

## Abstract

To date, no large scale, systematic description of the blood serum proteome has been performed in inflammatory bowel disease (IBD) patients. By using microarray technology, a more complete description of the blood proteome of IBD patients is feasible. It may help to achieve a better understanding of the disease. We analyzed blood serum profiles of 1128 proteins in IBD patients of European descent (84 Crohn’s Disease (CD) subjects and 88 Ulcerative Colitis (UC) subjects) as well as 15 healthy control subjects, and linked protein variability to patient age (all cohorts) and genetic components (genotype data generated from CD patients). We discovered new, previously unreported aging-associated proteomic traits (such as serum Albumin level), confirmed previously reported results from different tissues (i.e., upregulation of APOE with aging), and found loss of regulation of MMP7 in CD patients. In carrying out a genome wide genotype-protein association study (proteomic Quantitative Trait Loci, pQTL) within the CD patients, we identified 41 distinct proteomic traits influenced by cis pQTLs (underlying SNPs are referred to as pSNPs). Significant overlaps between pQTLs and cis eQTLs corresponding to the same gene were observed and in some cases the QTL were related to inflammatory disease susceptibility. Importantly, we discovered that serum protein levels of MST1 (Macrophage Stimulating 1) were regulated by SNP rs3197999 (p = 5.96E-10, FDR<5%), an accepted GWAS locus for IBD. Filling the knowledge gap of molecular mechanisms between GWAS hits and disease susceptibility requires systematically dissecting the impact of the locus at the cell, mRNA expression, and protein levels. The technology and analysis tools that are now available for large-scale molecular studies can elucidate how alterations in the proteome driven by genetic polymorphisms cause or provide protection against disease. Herein, we demonstrated this directly by integrating proteomic and pQTLs with existing GWAS, mRNA expression, and eQTL datasets to provide insights into the biological processes underlying IBD and pinpoint causal genetic variants along with their downstream molecular consequences.

## Author Summary

GWAS have resulted in greater than one hundred susceptibility loci for inflammatory bowel disease (Crohn’s Disease and Ulcerative Colitis). However, the molecular etiology of these diseases is not completely understood. In this study we profiled serum protein levels in IBD and control subjects and demonstrated an association of the levels of some proteins to Crohn’s Disease (CD) as well as aging. For the first time, we report proteomic QTLs (pQTLs) among CD patients, identifying proteomic traits corresponding to 41 distinct genes that were significantly influenced by SNP genotypes in cis. Particularly, we found that a well-known IBD risk locus on chromosome 3 is associated with significant changes of Macrophage Stimulating 1 (MST1) protein levels. As this result is consistent with MST1 eQTLs in liver and adipose tissues (but not whole blood), we believe that one possible mechanism of action of this genetic polymorphism alters expression and translation of MST1 in certain tissues (e.g. liver and adipose), which in turn results in changes of serum levels of the MST1 protein, and ultimately leading to increased risk of IBD.

## Introduction

The study of molecular mechanisms is of great importance for understanding the etiology of disease. Genome wide association studies (GWAS) help to identify genetic loci that are likely to contain causal variants for human diseases. Investigation of molecular phenotypes and how they relate to disease susceptibility can help close the gap in understanding between variations in the human genome that associate with disease and the biological processes that lead to disease. The integration of these two lines of research has proven particularly fruitful with the availability of high-throughput technologies (e.g., microarray and RNASeq), which allow for the measurement of the expression of genes comprising the entire transcriptome simultaneously across populations of individuals.

Circulating protein levels are known to be an important readout for diagnosing disease and tracking disease progression. Nevertheless, only recently have researchers begun employing high-throughput screening technologies to measure circulating protein levels in large human populations [1–4]. In this study, we employed a microarray technology (SOMAscan, Materials and Methods) to assess variations in the levels of 1128 proteins in the blood serum of three cohorts representing different disease conditions: Crohn’s Disease (CD, n = 84), Ulcerative Colitis (UC, n = 88) and Normal Controls (NC, n = 15). Descriptive summaries of the study cohorts with respect to age, sex and disease condition are reported in S1 Table. Molecular impact of aging has been extensively studied at the epigenetic [5] and transcriptome level [6]. However, the high throughput proteome aging profile has only been studied in healthy subjects [4]. We attempted to close this gap by computing for the first time the aging profile of UC and CD patients, and comparing them with their normal counterpart. Further, we generated genome-wide genotype data (12.6 million SNPs, assayed and imputed) on the CD patients and systematically characterized the genetic variance component for each of the serum proteomic traits (proteomic quantitative trait loci, pQTL).

## Results

### A serum proteomics aging signature in IBD patients

We studied the relationship between age and expression levels for 1128 proteins measured in the serum of the 15 NC individuals (all between 39 and 62 years old), 88 UC patients (all between 18 and 77 years old), and 84 CD patients (all between 18 and 64 years old). A normal linear regression was performed for each probe representing each protein, using the log2-transformed probe intensity as the outcome variable. Sex, batch, and time point were included as covariates (Materials and Methods).

At a 10% false discovery rate (FDR), we observed no proteomic traits in NC, 32 in CD (16 positive and 16 negative), and 130 traits in UC (87 positive and 43 negative) associated with age (S2 Table). The lack of a significant aging signature in NC could mainly be attributed to both a small sample size and the reduced age range in the subjects’ age. We detected fewer age-associated traits in CD patients compared to UC patients (S1 Fig), despite similar sample size in the two disease groups. Similar differences were observed for sex-associated traits in CD and UC (S1 Fig). Because CD and UC subjects were assayed on different SomaSCAN plates, we were not able to determine whether fewer genes were influenced by age and sex in CD than in UC, due to batch effect, or both. A more definite answer would require further investigation with adequate study design.

**Fig 1:**
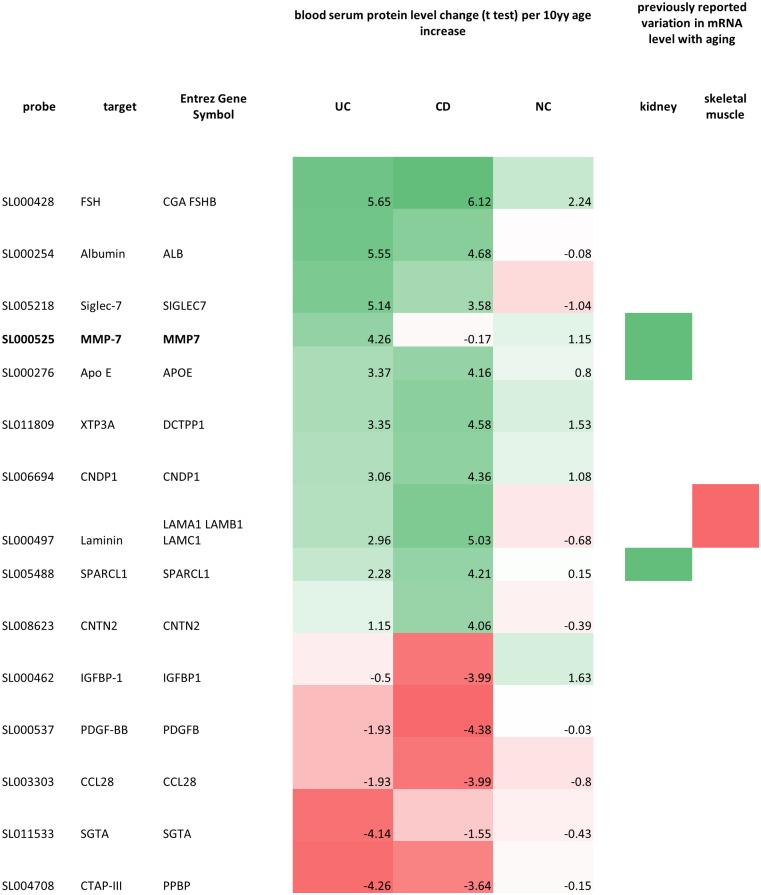
Heatmap of top significant age-related proteins. Included in the heatmap are all microarray probes with pvalue ≤ 1E-4 in at least one cohort. The UC, CD and NC cells are color-coded according to the Wald t-test of the age coefficient of the protein levels in each cohort, with the t test further reported in each cell. The last 2 columns report previously known association of gene mRNA levels with age within different tissues. Shades of green indicate increase of protein or mRNA level with aging; shades of red indicate decrease of protein or mRNA level with aging; the white color indicates lack of evidence in either direction.

The serum proteomic traits most strongly associated with age in UC, CD, and NC are depicted as a heatmap in Fig 1 (p ≤ 1E-4 in at least one cohort), alongside previously reported proteomic results from kidney [7] and skeletal muscle [8]. We observed generally good agreement of results among all three cohorts, despite the limited sample sizes. We further intersected our aging signatures with a proteomics aging signature derived from a study of healthy individuals [4] in which only the top 10 significant results were released (Table 1). The overlap was significant in CD (OR = 6.48, p = 0.011) and UC (OR = 6.29, p = 0.006). Only one gene from previous published proteomics aging signature [4] was confirmed in our healthy cohort, CHRDL1 and not statistically significant (OR = 2.84, p = 0.323). We conducted gene set enrichment analysis (GSEA) on 23 MSigDB curated gene sets related to aging (S3 Table). At a 10% FDR, 2 gene sets were positively enriched in UC: “LEE AGING CEREBELLUM UP” and “DEMAGALHAES AGING UP”. Positive enrichment of the 2 gene sets was also observed in NC, though none reached statistical significance. No gene set showed significant enrichment in CD at a 10% FDR.

**Table 1:** Overlap of proteomic aging signature between study samples and previous report.

Cohort	Size of Aging Signature*	Overlap Genes**	Overlap odds ratio	Overlap pvalue
UC	221	CCDC80, CHRDL1, CST3, FSTL3, ROR1, THBS4	6.29	0.0055
CD	108	CCDC80, FSTL3, IGFBP6, THBS4	6.48	0.0106
NC	43	CHRDL1	2.84	0.3231

* Number of proteomic traits with nominal pvalue≤0.05 of positive association with aging.

**Genes overlapping with aging signature reported by Menni et al [4]; all 10 genes in Menni et al [4] were reported as having a positive association with aging.

**Fig 2:**
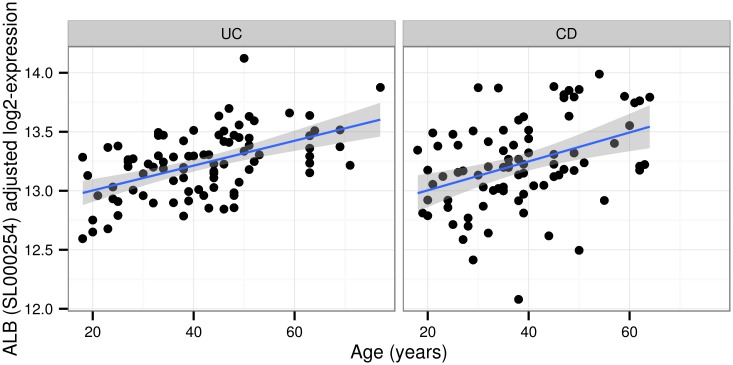
Increase of Albumin levels with aging in CD and UC. Scatterplot of the Albumin protein level vs patients age, separately for UC patients (left panel) and CD patients (baseline data only is displayed, right panel). Age in years on the horizontal axis; mean-centered and adjusted log2-protein expression on the vertical axis (adjusted for sex and plateID).

**Fig 3:**
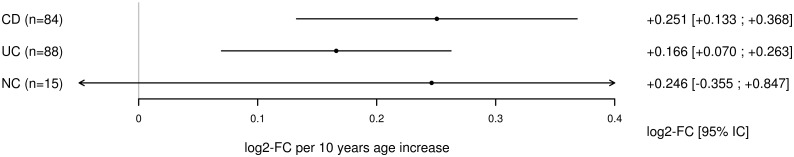
Variation of APOE levels with aging in CD, UC and NC subjects. Forest plot of the estimated log2-FC of APOE protein levels (probe SL000276) per 10 years increase in age, with 95% confidence intervals, as obtained from the differential expression analysis performed separately in Crohn’s Disease (CD), Ulcerative Colitis (UC) and Normal Controls (NC) subjects. Estimated log2-FCs and 95% Confidence Intervals are further reported on the right.

Interestingly, both UC and CD patients displayed a slow but consistent increase in Albumin levels with age (Fig 2). The estimated log2 fold change (log_2_FC) per 10 years increase was 0.11 (SE = 0.02) in UC, and 0.12 (SE = 0.03) in CD. We found that APOE was upregulated in the blood serum of older subjects, in agreement with previous reports on the APOE mRNA levels in kidney [7]. APOE is known for its role in arteriosclerosis, Alzheimer’s disease, Parkinson’s disease and cardiovascular diseases [9]. The positive association (i.e., log_2_FC estimates) between serum APOE levels and age was fairly consistent across cohorts with different disease conditions. The increase in APOE concentration was mostly pronounced in CD and NC subjects, with its levels roughly doubling in 40 years (Fig 3).

As previously reported in kidney [7], we observed upregulation of matrix metalloproteinase-7 (MMP7) with aging among the UC patients (log_2_FC = 0.09, SE = 0.02, p = 5.31E-5). However, association between age and MMP7 was absent within the CD patients (log_2_FC = -0.01, SE = 0.03, p = 0.866) and non-significant in the healthy controls (log_2_FC = 0.15, SE = 0.13, p = 0.2756). A Cochran’s Q test of heterogeneity between the estimates obtained from our 3 cohorts was significant (Q = 7.16, p = 0.0278), suggesting that the observed differences might not be attributed to sampling variability alone. MMP7 breaks down the extracellular matrix not only during embryonic development, reproduction, and tissue remodeling, but in disease processes such as arthritis as well. MMP7 is also known to be involved in inflammation and wound healing. In mice studies, MMP7 has been shown to regulate the intestinal bacterial microbiome, and is thus an important gene for the immune response and homeostasis in the intestine [10]. Chronic stress on the immune system among CD patients may disrupt the slow increase of MMP7 levels with increasing age.

### Genetics of proteomic traits in serum

We performed proteomic-QTL mapping in 51 Caucasian CD subjects for which genome-wide genotype data were available. Because proteomic profiling was carried out on each CD patient at two time points, baseline and after 22 weeks, there were a total of 102 samples used in the analysis. A statistical approach similar to eQTL mapping was employed (Materials and Methods). At a 10% FDR, cis pQTL for 41 proteomic traits were mapped (Table 2). A full list of pQTLs at a 50% FDR was provided in S4 Table.

**Table 2:** Newly discovered protein-QTLs (10% FDR): protein probe and lead significant SNP.

*probeID*	*Target*	*UniProt*	*gene*	*rsID*^1^	*chr*	*pos (Mb)*^2^	*alleles*^3^	*direction*^4^	*pvalue*^4^
SL008063	PPAC	P24666	ACP1	rs17713879	2	0.25	A/G	increase	2.46E-10
SL008039	AK1A1	P14550	AKR1A1	rs6688727	1	46.00	A/G	increase	2.92E-06
SL000003	Angiogenin	P03950	ANG	rs72659950	14	21.12	A/G	increase	4.79E-06
SL010375	ASAH2	Q9NR71	ASAH2	rs137889787	10	51.99	A/G	decrease	1.18E-09
SL008644	BST1	Q10588	BST1	rs73241195	4	15.66	C/G		6.19E-13
SL003190	MIP-5	Q16663	CCL15	rs854685	17	34.32	A/G	increase	3.44E-07
SL003197	TECK	O15444	CCL25	rs184230513	19	8.12	A/G	increase	3.66E-10
SL000516	MCP-3	P80098	CCL7	rs1133763	17	32.65	A/C	increase	8.76E-10
SL005215	Siglec-3	P20138	CD33	rs12459419	19	51.73	A/G	decrease	4.02E-11
SL006029	Chitotriosidase-1	Q13231	CHIT1	rs2244783	1	203.19	A/G	decrease	1.19E-11
SL006694	CNDP1	Q96KN2	CNDP1	rs4329999	18	72.23	A/G	decrease	4.09E-06
SL014048	CONA1	Q86Y22	COL23A1	rs2133281	5	177.89	A/G	decrease	8.98E-07
SL004015	TAFI	Q96IY4	CPB2	rs9526134	13	46.62	A/C	increase	4.69E-06
SL011808	CPNE1	Q99829	CPNE1	rs143646210	20	34.20	A/G	decrease	1.60E-07
SL010456	CYTN	P01037	CST1	rs4260306	20	23.73	A/G	decrease	7.33E-16
SL004673	Cathepsin S	P25774	CTSS	rs72702561	1	150.69	A/G	increase	2.37E-06
SL006550	ECM1	Q16610	ECM1	rs77257036	1	150.48	A/G	decrease	4.01E-08
SL000408	Epo	P01588	EPO	rs73170784	7	100.36	A/G	increase	1.22E-06
SL007729	ARTS1	Q9NZ08	ERAP1	rs13178387	5	96.14	A/C	decrease	4.63E-09
SL012469	GPC5	P78333	GPC5	rs1332090	13	92.45	A/G	increase	7.00E-08
SL006512	HGFA	Q04756	HGFAC	rs2857985	4	3.48	A/G	increase	6.71E-11
SL000437	Haptoglobin Mixed Type	P00738	HP	rs12925078	16	72.07	A/G	decrease	2.84E-06
SL002922	sICAM-1	P05362	ICAM1	rs901886	19	10.40	A/G	increase	1.66E-15
SL010368	IDUA	P35475	IDUA	rs11248061	4	0.98	A/C	increase	9.54E-06
SL001943	IL-6 sRa	P08887	IL6R	rs12133641	1	154.43	A/G	decrease	3.57E-11
SL003184	sLeptin R	P48357	LEPR	rs61781312	1	66.09	A/G	increase	6.78E-30
SL005190	ILT-2	Q8NHL6	LILRB1	rs10427127	19	55.14	A/G	increase	5.17E-14
SL005191	ILT-4	Q8N423	LILRB2	rs386056	19	54.78	A/G	decrease	1.39E-06
SL000510	Lysozyme	P61626	LYZ	rs1384	12	69.75	A/G	decrease	1.09E-07
SL004516	MBL	P11226	MBL2	rs10824792	10	54.53	A/G	increase	1.57E-09
SL005202	MSP	P26927	MST1	rs9836291	3	49.70	A/G	decrease	1.79E-11
SL008611	ASAHL	Q02083	NAAA	rs3796484	4	76.87	A/T		7.86E-06
SL005212	Prolactin Receptor	P16471	PRLR	rs34623800	5	35.01	A/G	decrease	3.55E-06
SL000572	SAA	P0DJI8	SAA1	rs11024616	11	18.33	C/G		1.12E-06
SL010470	Semaphorin 3E	O15041	SEMA3E	rs2191530	7	83.15	A/G	increase	6.65E-07
SL014292	SIG14	Q08ET2	SIGLEC14	rs73052804	19	52.16	A/G	decrease	2.20E-08
SL005219	Siglec-9	Q9Y336	SIGLEC9	rs2075803	19	51.63	A/G	increase	4.92E-27
SL016928	SLAF7	Q9NQ25	SLAMF7	rs2295616	1	160.71	A/G	decrease	2.20E-06
SL003043	TIMP-3	P35625	TIMP3	rs5754272	22	33.17	A/G	increase	1.77E-09
SL003198	Tenascin	P24821	TNC	rs11787994	9	117.75	A/C	increase	5.80E-06
SL004782	TSG-6	P98066	TNFAIP6	rs73004003	2	152.17	A/G	increase	3.81E-06

^1)^ Lead pSNP;

^2)^ Hg19;

^3)^ effective allele/alternative allele;

^4)^ estimated effect of the effective allele on the protein expression level

We explored the concordance between serum pQTL and eQTLs in various tissues (Table 3). Interestingly, serum pQTLs and whole blood eQTLs did not overlap more than would be expected by chance, whereas liver eQTL significantly overlapped with serum pQTLs (fold enrichment = 2.33, p = 5.31E-5). Proteins circulating in blood represent peptides from many tissues, with liver, but not blood lymphocytes, representing one of the primary sources of circulating serum proteins. Further, transcriptome profiling in blood is not a close surrogate of serum proteomics (Table 3). Thus, pQTLs carry orthogonal information not captured by mRNA/eQTL and thus have the potential to provide unique insights into the molecular etiology of IBD and other diseases.

**Table 3:** Overlaps between blood serum protein-QTLs and previously published eQTLs from several tissues (10% FDR).

Tissue	observed overlap^1^	expected overlap^2^	fold enrichment	Fisher test pvalue
Blood[12]	14	14.2	0.99	1.000
Cerebellum^3^	9	7.4	1.22	0.535
Prefrontal^3^ Cortex	8	7.9	1.01	1.000
Visual Cortex^3^	4	4.9	0.82	0.809
Liver[41]	20	8.6	2.33	0.000
Omental fat [41]	16	14.2	1.13	0.616
Subcutaneous fat [41]	15	12.2	1.23	0.383

^1)^ number of shared gene symbols;

^2)^ expected number of shared gene symbols under the assumption of independence between the two studies; a list of 991 gene symbols which were tested for cis-eQTLs in both the present study and any of the eQTL studies was used as a background;

^3)^ eQTLs from the Harvard Brain collection (www.brainbank.mclean.org).

#### Serum pQTLs were enriched for GWAS loci of IBD and inflammatory diseases

**Fig 4:**
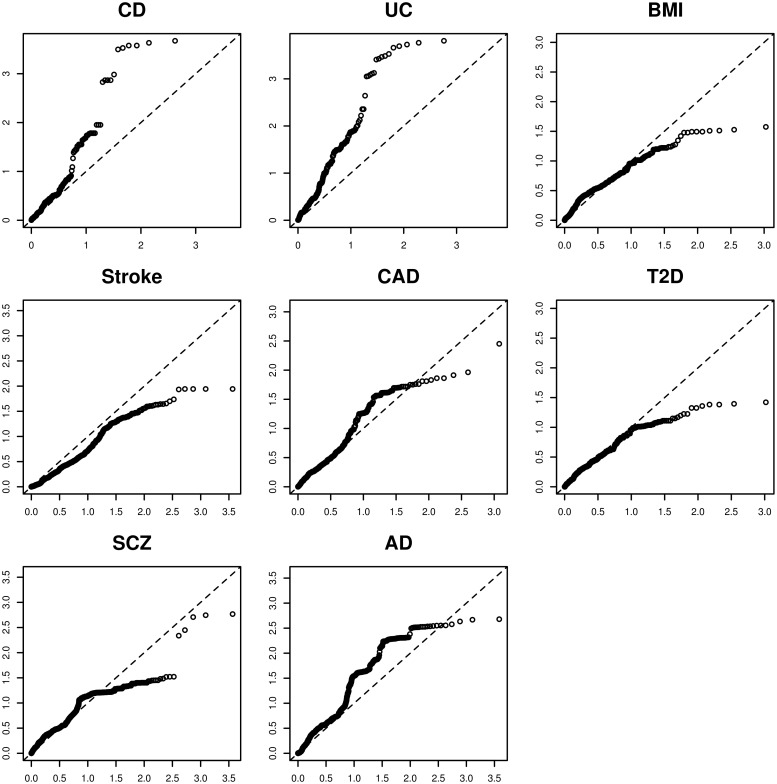
Enrichment for GWAS signals in blood protein-QTLs of CD patients. Expected uniform -log10(relative rank) of the protein-SNPs (nominal pvalue ≤ 1E-5) within the full GWAS SNPs list on the horizontal axis; observed–log10(relative rank) on the vertical axis. CD: Crohn’s Disease; UC: Ulcerative Colitis; BMI: Body Mass Index; SCZ: Schizofrenia; Stroke: Ischemic Stroke; T2D: Type-2 Diabetes. References for all the studies are reported in the Methods section.

It is well established that eSNPs are significantly enriched for GWAS SNPs [11, 12]. To explore whether pSNPs were also enriched for GWAS human disease SNPs, we inspected the ranks of the pSNPs within published GWAS (Materials and Methods) to test whether pSNPs were enriched for small GWAS p values (Fig 4). Interestingly, while serum pSNPs were enriched for CD and UC GWAS SNPs [13], they were not enriched for other disease associated traits or diseases such as Body Mass Index (BMI) [14], Schizophrenia (SCZ) [15], Ischemic Stroke (Stroke) [16] and Type-2 Diabetes (T2D) [17]. This specificity for IBD GWAS may be attributed to both the study cohort (CD patients) as well as to the tissue’s relevance to the disease. The significant enrichment of pSNPs for UC and CD GWAS SNPs highlights the potential utility of pSNPs for elucidating IBD etiology.

We conducted gene set enrichment analysis using MetaCore from Thomson Reuters (https://portal.genego.com) and found that 12 out of 41 pQTLs were involved in inflammatory response: CCL7, CCL15, CCL25, ECM1, EPO, HP, ICAM1, IL6R, LYZ, MBL2, SAA1 and TNFAIP6. CD157, also known as ADP-ribosyl cyclase 2, is coded by the BST1 gene. CD157 serum levels were significantly influenced by pSNPs, which were documented as Parkinson’s Disease GWAS SNPs [18–22]. CD157 is part of a supramolecular complex with CD11b/CD18 on the human neutrophil cell surface, and an important mediator of neutrophil adhesion and migration [23, 24]. BST1 expression is enhanced in bone marrow stromal cell lines derived from patients with rheumatoid arthritis. Further, the CD157 pQTL is coincident with the BST1 eQTL in whole blood [12](S2 Fig). IL-6 sRa, encoded by the IL6R gene, is significantly controlled by cis-pSNPs (p = 3.57E-11, Table 2 and S5 Table). GWAS’s have related IL6R to immune diseases and associated traits, including coronary heart disease [25], pulmonary function [26], asthma [27], C-reactive protein [27, 28], rheumatoid arthritis [29] and IBD susceptibility [30, 31]. The association of IL6R locus with CAD was genome significant reported Cardiogram study [32] (S5 Table). IL6R was also detected as an eQTL in whole blood [12]. Interestingly, effect direction was opposite for serum pQTL and blood eQTL (S5 Table). That is the CAD risk allele, G, decrease serum IL6R protein level, but increase IL6R mRNA level in blood cells.

Siglec-3, coded by the CD33 gene, is a transmembrane receptor expressed on cells of myeloid lineage [33], and its serum levels were strongly controlled by pQTL (p = 4.02E-11, Table 2). CD33 is an established susceptibility locus for Alzheimer’s disease [34–39], where the risk allele has been found to alter monocyte function and amyloid biology [36]. In the paper, we found CD33 serum level was influenced by Alzheimer’s disease GWAS SNP, where the risk allele, rs12459419-G, was associated with higher serum CD33 level. This suggesting rs12459419 may influence CD33 transcription, translation or post-translation control of CD33 product (Siglec-3), and in turn modify Alzheimer’s disease risk.

#### MST1 as a mediator of CD and UC risk

**Fig 5:**
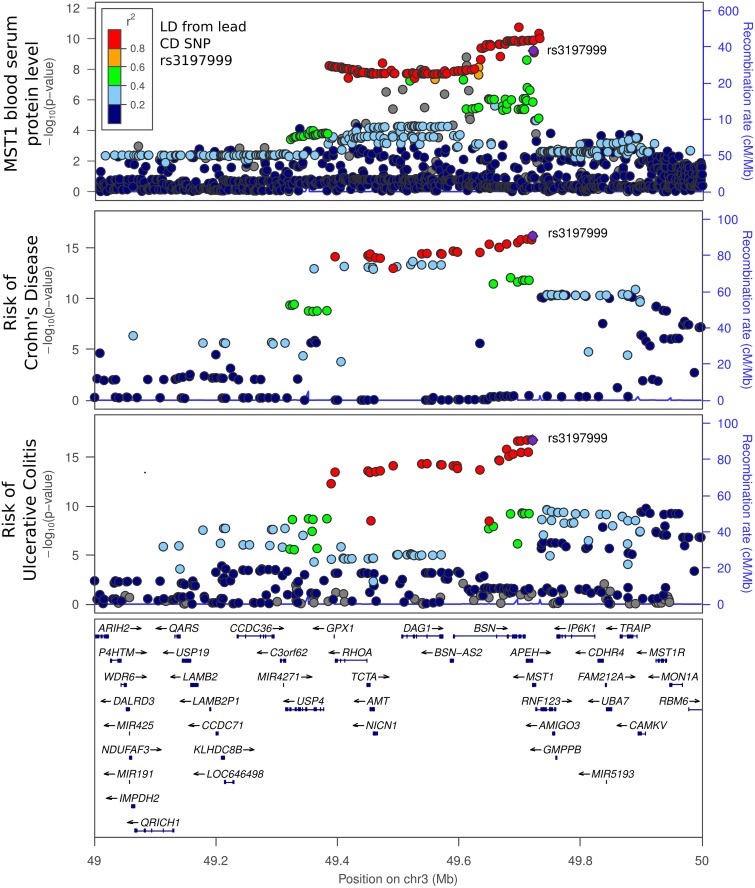
Locuszoom plot of MST1, CD and UC association pvalues around the MST1 gene. It is worth noting that our proteomics platform has 4 probes in this chromosomal region, targeting 4 different proteins: IMPDH2 (probe SL010928), MST1 (probe SL005202), MST1R (probe SL004637) and MAPKAPK3 (probe SL004765). Of these, MST1 is most significantly associated with the IBD GWAS SNP in this locus (S3 Fig), and the association pattern was highly consistent with the CD and UC GWAS peaks (Fig 5).

Our pQTL analysis revealed a chromosome 3 SNP (rs3197999), located within the MST1 (Macrophage Stimulating 1) gene, associated with MST1 protein levels (p = 5.96E-10). This locus is known to be associated with CD and UC susceptibility [13]. Prompted by this finding, we extended our pQTL analysis to fully cover the region chr3:48Mb-51Mb (S6 Table). The pattern of significance of association between genotype and serum MST1 levels matches closely that of association with CD and UC risk (Fig 5), a strong indication that MST1 protein levels and IBD share a common causal genomic variant.

The lead CD risk SNP in this region is rs3197999, a nonsynonymous mutation located within exon 18 of MST1. The minor allele ‘A’ is associated with an increased risk of both CD and UC (p = 6.20E-17 and 1.86E-17, respectively), and a decrease of MST1 protein levels (p = 5.96E-10). In our CD cohort, the risk allele ‘A’ has a frequency of 24.51%, which is in line with the observed frequency in the 1000 Genome Project CEU population (25.76%). A strong association of this SNP has also been reported with MST1 mRNA levels in liver (p = 7.65E-10) and subcutaneous fat (p = 1.20E-7), although interestingly not in blood, again demonstrating that peptides circulating in blood can reflect activity levels or abundance of different tissues other than blood. GTex data showed MST1 expression was 31.7 fold higher in liver compared to the average of all other tissues (S3 Fig).

## Discussion

GWAS analysis has identified more than one hundred genome-wide significant loci for IBD [13, 40]. Systems biology approaches (e.g. eQTLs and gene networks) have been used to fill the knowledge gap between GWAS SNPs and IBD susceptibility. However, most of these analyses have been applied at the mRNA expression level. Today, the technology and analytic tools are in place for large scale proteomic analysis in IBD relevant tissues. In this report, we leverage the high throughput analysis of the serum proteome to provide insight into the molecular etiology of IBD, and reveal the possible mechanisms of GWAS SNPs. Novel insights into the biology of disease can be missed if analyzing at the mRNA level or by low throughput protein analysis. Our results argue for the importance of a large-scale systems biology study of the proteome space to reveal the complete picture of molecular level alteration and disease predisposition in IBD.

We observed a large degree of overlap between the aging signatures from our 3 discovery cohorts (Fig 1) and between our signatures and one previously published from healthy individuals by Menni et al [4]. This suggests that the circulating blood proteome has a robust aging pattern which is consistent across populations of diverse disease conditions. For example, the concentration of albumin, which constitutes a large fraction of the blood serum protein contents, increases slowly but consistently with aging of CD and UC patients. In contrast, we observed positive association between age and MMP7 (matrix metalloproteinase-7) levels in UC and normal controls, but this association was markedly absent in CD patients. MMP7 is known to be involved in inflammation and wound healing. The loss of age-MMP7 association among CD patients may reflect the disease progression, in other words, chronic stress on the immune system among CD patients may disrupt and slow the increase of serum MMP7 levels with aging. In this study, we employed multiple SomaSCAN plates to assay all specimens, with CD, UC and normal control subjects assayed in different plates at different time points. The proteomic profile showed systematic differences among the plates (S6 and S7 Figs). From the Principal Components Analysis we see separation between the different disease groups. However it is challenging to distinguish whether differences observed were due to batch effects or to true biological differences between UC, CD and normal subjects. This design problem prevented us from directly studying the serum proteomic signature of UC and CD. Instead, we investigated age- and sex-associated genes within CD, UC and normal controls.

To our knowledge, this study is the first systematic mapping of proteomic QTLs in a cohort of Crohn’s Disease patients. At 10% FDR, we found 41 distinct proteins showing evidence of association with a nearby (cis) SNP. Some of these genes and loci were previously discussed in relation to diseases and other molecular QTL studies, such as BST1, a gene known to be implicated in Parkinson’s Disease [18–20].

Many of our 10% FDR pQTL results were previously reported as eQTLs in various tissues. However, overlap between our blood serum pQTLs results and mRNA eQTLs derived from several large tissue sets (including whole blood) was not higher than random chance (Table 3). Interestingly, liver eQTLs showed significant overlap with serum pQTLs. In the present study we screened protein products circulating in the blood serum as opposed to mRNA extraction from cells in solid and soft tissues biopsies, such as liver, fat and brain sections. Said otherwise, the blood serum proteome captures secretions from multiple and distant tissues and cell types, and thus observations from blood serum are to be expected to depart from those done in studies focused on the mRNA levels of a single tissue or cell type, and contain substantial molecular information not otherwise covered by mRNA surveys.

We further systematically surveyed for the presence of eQTLs and/or pQTLs among known IBD risk loci collected from the NHGRI-EBI GWAS catalog [42]. In particular, we examined eQTL of blood [12], brain (Harvard Brain collection, www.brainbank.mclean.org), liver [41], omental fat [41] and subcutaneous fat [41] tissues. Out of 393 published IBD risk loci, 149 were not eQTLs nor pQTLs for any of the surveyed tissues, 241 were eQTLs for one or more tissues, and 3 were both eQTLs and pQTLs (all 3 in the MST1 locus). Full results of our survey, SNP by SNP, are reported in S9 Table.

In this paper, we demonstrated the potential of pQTLs as a powerful tool to interpret GWAS findings. Crohn's disease and ulcerative colitis susceptibility has been mapped to a wide locus of 3p21. Possible genes underlying this GWAS locus include BSN (bassoon), MST1 (macrophage stimulating-1), MST1R (MST1 Receptor), etc [43]. The lead GWAS SNP, rs3197999, is associated with the gene expression level of many genes in various tissues (e.g. UBA7 and HPEH in blood, CAPN5 and RBM6 in adipose, and MST1 in liver and adipose tissues) [12, 41]. MST1 gene encoding Macrophage Stimulating Protein (MSP), and MSP binding to the MSP receptor (also known as RON receptor). The rs3197999 SNP results in an Arg689Cys amino acid substitution within the β-chain of MSP (MSPβ) [44]. Therefore, rs3197999 (MSPβ Arg689Cys) can possibly function by at least two mechanisms, (1) affecting the protein structure and function; and (2) regulating the protein levels in vivo.

Evidence of MSPβ Arg689Cys’s effect on protein function remains inconsistent to date. Gorlatova et al. showed MSPβ Cys689 (GWAS risk allele) binding affinity to RON is approximately 10-fold lower than that of the wild-type MSPβ (Arg689) [44]. However, in a eukaryotic cell system, the Cys689 allele significantly increased the stimulatory effect of MSP on chemotaxis and proliferation by THP-1 cells, indicating a gain of function associated with the Cys689 allele [45]. In this study, we pointed out another possible mechanism that the GWAS SNP (rs3197999) causes IBD by regulating protein level of MSP. Shown in S8 Fig, the risk allele (rs3197999-A which codes Cys689) profoundly decreases serum MSP level (p = 5.96e-10). It is unclear whether lower serum MSP contributes to IBD risk, but it is reported that MST1R expression was significantly downregulated in other immune disease (ie, multiple sclerosis) in both mouse and human subjects [43]. We also noticed that the MST1 pQTL peak is almost identical to the IBD GWAS peak in the 3p21 locus in terms of location and shape, despite the pQTL and GWAS studies being carried out in completely independent cohorts (Fig 5). In this study, we measured several additional proteins on 3p21 locus with the SomaSCAN platform (IMDH2, MSP R and MAPKAPK3), but none of them showed pQTLs (S3 Fig). Furthermore, the MST1 eQTL and MSP pQTL are consistent in direction, where the risk allele (rs3197999-A) is linked to lower MST1 expression and lower MSP serum level. It is possible that results of SOMAscan can be affected by non-synonymous mutations. Although the exact binding site of MST1 Somascan probe is not known, distinct +/- binding of the MST1 probe on sample groups were not observed on Somalogic Inc development/validation samples, indicating at least the non-synonymous variant does not have a profound impact on the probe binding properties. In parallel, association of the rs3197999 risk allele with lower MST1 protein concentration in serum was also recently reported in a cohort of 4900 healthy individuals from the Gutenberg Health Study using ELISA assay [46], which further corroborates the reproducibility of our results. Taken together, our data suggest that the IBD locus 3p21 is attributable to the MST1 gene, and the possible mechanism is that the risk allele reduces MST1 mRNA abundance in relevant tissues as well as MSP protein level. The lower MSP in turn modify macrophage activities and lead to IBD risk.

## Materials and Methods

### Subjects

Blood serum proteomics profiles were available for 15 normal controls (NC) between 39 and 62 years old. Serum samples were available from the baseline pre-treatment visit of 88 Ulcerative Colitis (UC) patients between 18 and 77 years old who were enrolled in the PURSUIT study [47], as well as baseline and 22 weeks follow up visits of 84 moderate to severe Crohn's Disease (CD) patients between 18 and 64 years old who were enrolled in the CERTIFI study [48]. All subjects were of Caucasian ancestry (self reportedly).

### Proteomics

Proteins were measured using a SOMAmer-based capture array called “SOMAscan” [2, 49] (web site: http://www.somalogic.com/Products-Services/SOMAscan). A total of 1,128 proteins were measured by an approach that uses chemically modified nucleotides to convert a protein signal to a nucleotide signal that is measured as relative fluorescence units using a custom DNA microarray.

### Genotyping

Genotyping of CD subjects was performed at the Medical Genetics Institute as Cedars-Sinai Medical Center using Illumina OmniExpress chips (Human610-Quadv1 Chips; Illumina, San Diego, CA, USA). Genotypes were determined based on clustering of the raw intensity data for the two dyes using Illumina BeadStudio software. Six samples performed in duplicate yielded >99% concordance. In total, 733'120 SNPs were successfully genotyped. Genotype imputation was performed using the 1000G reference following the MaCH pipeline [50].

### Differential protein expression analysis

Differential protein expression analysis was performed by linear regression models, using the log-2 transformed protein level as the outcome variable (y) and age plus other covariates as regressors.

Specifically, the following ordinary least squares regression was performed in UC and NC: y ~ Age + Sex + PlateID. Within the CD cohort, as two separate measures were available from two different time points, a mixed effects model was estimated: y ~ Age + Sex + PlateID + TimePoint + (1|SubjectID), where '1|SubjectID' represents the random intercept associated with each CD subject. In all cases, significance of the association with Age was quantified with the two-sided Wald test on the 'Age' coefficient.

We estimated the False Discovery Rate using a previously reported empirical permutation approach [51–53], and N = 1000 permutation iterations were run. Specifically, FDR was computed for each probe as:

$$\hat{FDR}\left(t\right)=\text{min}(1,\frac{avg\left(\#\;permuted\;pvalues\leq t\right)}{observed\;pvalues\leq t})$$

### Gene set enrichment analysis

Gene Set Enrichment Analysis of differential expression results was performed using the GSEA software from the BROAD institute, v2.2.0, and the MSigDB c2 (curated gene signatures) Gene sets database, gene symbols, v5.0 (http://software.broadinstitute.org/gsea). Results from each cohort were analyzed separately, using the 'preranked gene list' method. False Discovery Rate was evaluated by running 1000 permutations.

### Proteomic-QTL mapping

We performed proteomic-QTL mapping on 51 Caucasian CD subjects with available imputed genotype data. A total of 102 samples were finally available for the analysis (all subjects had 2 proteomics assays available, at baseline and at 22 weeks follow up).

A random effects linear regression model was adopted to map cis protein-QTLs (pQTLs): y ~ EffectiveAlleleCopyNumber + Age + Sex + TimePoint + (1|SubjectID), where 'y' is the inverse-normal transformed protein expression level, 'EffectiveAlleleCopyNumber' is the imputed allele copy number for a specific SNP, and '1|SubjectID' represents the random intercept associated with each CD subject. Significance of the genotype effect was quantified with a two-sided Wald test on the Maximum Likelihood estimator of its coefficient. The distribution of the Wald test pvalue across all cis effects under the null hypothesis of no correlation between genotype and gene expression was estimated by re-running the analysis on a null dataset obtained by permuting the genotype subject identifiers. A self-contained, re-usable R script was written to fit the random effects models using the ‘lme4’ R package. The full code is available at github.com/antoniofabio/eqtl-ranef. FDR was quantified by comparing the observed distribution of the test statistic with that estimated from the permuted data, as previously described [51–53].

Additional regressions were run for probe SL005202 (gene symbol: MST1) against all SNPs in chromosome 3, between 49 and 51 mega-bases (hg19), conditioning first on the peak pSNP rs9836291 (chr3:49697459) and then on the IBD risk SNP rs3197999 (chr3:49721532), in addition to the covariates already used for the main model.

### Enrichment for GWAS signals in lists of SNPs

Enrichment for GWAS signals in proteomic-QTL hits was assessed as follows. First, full GWAS results (variants positions and pvalues) were retrieved from their original publications: Chron’s Disease and Ulcerative Colitis (CD and UC, [13]), Body Mass Index (BMI, [14]), Schizophrenia (SCZ, [15]), Ischemic Stroke (Stroke, [16]), and Type-2 Diabetes (T2D, [17]). The full GWAS tables were then reduced to the subset of SNPs covered by our pQTL study. Within each reduced table, the relative rank of the pvalue of each SNP was computed (e.g., in a table of 1E5 SNPs, the smallest pvalue has relative rank 1E-5, the second smallest has relative rank 2E-5, etc.). Finally, we plotted the relative ranks of our protein-SNPs within each table, and compared it with a uniform distribution using a rank-rank plot.

### Ethics statement

The current study is approved by the Icahn School of Medicine at Mount Sinai IRB with the approval number HSM11-01669, The study is also listed at ClinicalTrials.gov with reference number NCT00771667, and the protocol was approved by the institutional review board at each study center. All the participants received written consent forms.

## Supporting Information

S1 Fig Number of discoveries (vertical axis) by cohort (line colors) and model covariate (panels). UC dominates CD and NC for both Age (left panel) and Sex (right panel).
(PNG)

S2 Fig Association pvalues between SNPs in the chr4:15.25Mb-16Mb region and BST1 molecular traits: whole blood mRNA (published data, Westra et al., 2013) and blood serum protein levels (present study, probe SL008644). (PNG)

S3 Fig pQTL association pvalues of SNPs in the chr3:48Mb-51Mb region, and probes therein. (PNG)

S4 Fig Expression of MST1 across different human tissues. Image retrieved from GeneCards (www.genecards.org). It shows data pooled from BioGPS (biogps.org), GTex (www.gtexportal.org), and SAGE (cgap.nci.nih.gov/SAGE).
(PNG)

S5 Fig Histograms of age absolute effect sizes and probe intensity coefficients of variations in CD and UC. Difference in median absolute effect sizes between the two cohorts is not significant (Wilcoxon test p = 0.188). Difference in the coefficient of variation (SD/mean) is significant (Wilcoxon test p = 1.32e-14).
(TIFF)

S6 Fig Significance of batch effect on proteomics probe intensity. Qqplot showing -log10(pvalue)s expected under the null hypothesis of no batch effect (horizontal axis) and observed Kruskal-Wallis test pvalues of batch effect (vertical axis); each circle represents a single tested probe. For each probe, Kruskal-Wallis test was performed testing that the ‘location’ of the log-intensity of the probe was the same across the 5 available batches (271 samples, 4 degrees of freedom Kruskal-Wallis test).
(TIFF)

S7 Fig Array Data Principal Components by plate, disease status and time point. First two principal components (PC1 on the horizontal axis, PC2 on the vertical axis) with samples stratified by disease status (panel rows) and time point (panel columns) and color coded by array plate.
(PNG)

S8 Fig MST1 (probeID: SL005202) protein levels by rs3197999 genotype. (PNG)

S1 Table Study cohorts’ descriptive summaries. (XLSX)

S2 Table Aging differential protein expression analysis results in CD, UC and NC subjects. (XLSX)

S3 Table Gene Set Enrichment Analysis results of the aging signatures of CD, UC and NC subjects. (XLSX)

S4 Table Full, annotated cis-protein QTL results, up to FDR = 50%. (XLSX)

S5 Table Overlap between serum pQTLs and GWAS signal of genome-wide significance. (XLSX)

S6 Table MST1 proteomic-QTL results in the region chr3:49Mb-51Mb. Variants are annotated with MST1 association statistics, CD and UC risk statistics, rsIDs, gene and function (from annovar).
(XLSX)

S7 Table MST1 proteomic-QTL results in the region chr3:49Mb-51Mb, alternatively conditioning on the peak pSNP rs9836291 (chr3: 49697459) and on the IBD risk SNP rs3197999 (chr3:49721532). (XLSX)

S8 Table Distribution of baseline blood samples across microarray plates, by cohort and sex. (XLSX)

S9 Table Known IBD risk loci and 10% FDR mRNA expression-QTLs (eQTLs) and protein-QTLs (pQTLs) from different tissues. IBD risk loci were obtained from the NHGRI-EBI GWAS catalog (version 1.0.1 e84, 2016-06-12) and lifted to the hg19 genome build. For each locus, we surveyed 10% FDR cis or trans eQTL and pQTL studies from few tissues. Brain eQTLs (Prefrontal Cortex, Visual Cortex and Cerebellum) were obtained from the Harvard Brain collection (www.brainbank.mclean.org); Blood eQTLs from [12]; Liver, Omental fat and Subcutaneous fat from [41]; Blood serum pQTLs from the present study.
(XLSX)

S10 Table Protein expression summary statistics. Expression measured as log2-probe intensity.
(XLSX)

S11 Table Allele frequencies of all pSNPs with FDR ≤ 0.5. (XLSX)
