# Supplementary figures and images for: High-Throughput Characterization of Blood Serum Proteomics of IBD Patients with Respect to Aging and Genetic Factors
Source: PLoS Genet. 2017 Jan 27;13(1):e1006565. doi: 10.1371/journal.pgen.1006565 (PMC5271178; doi:10.1371/journal.pgen.1006565)

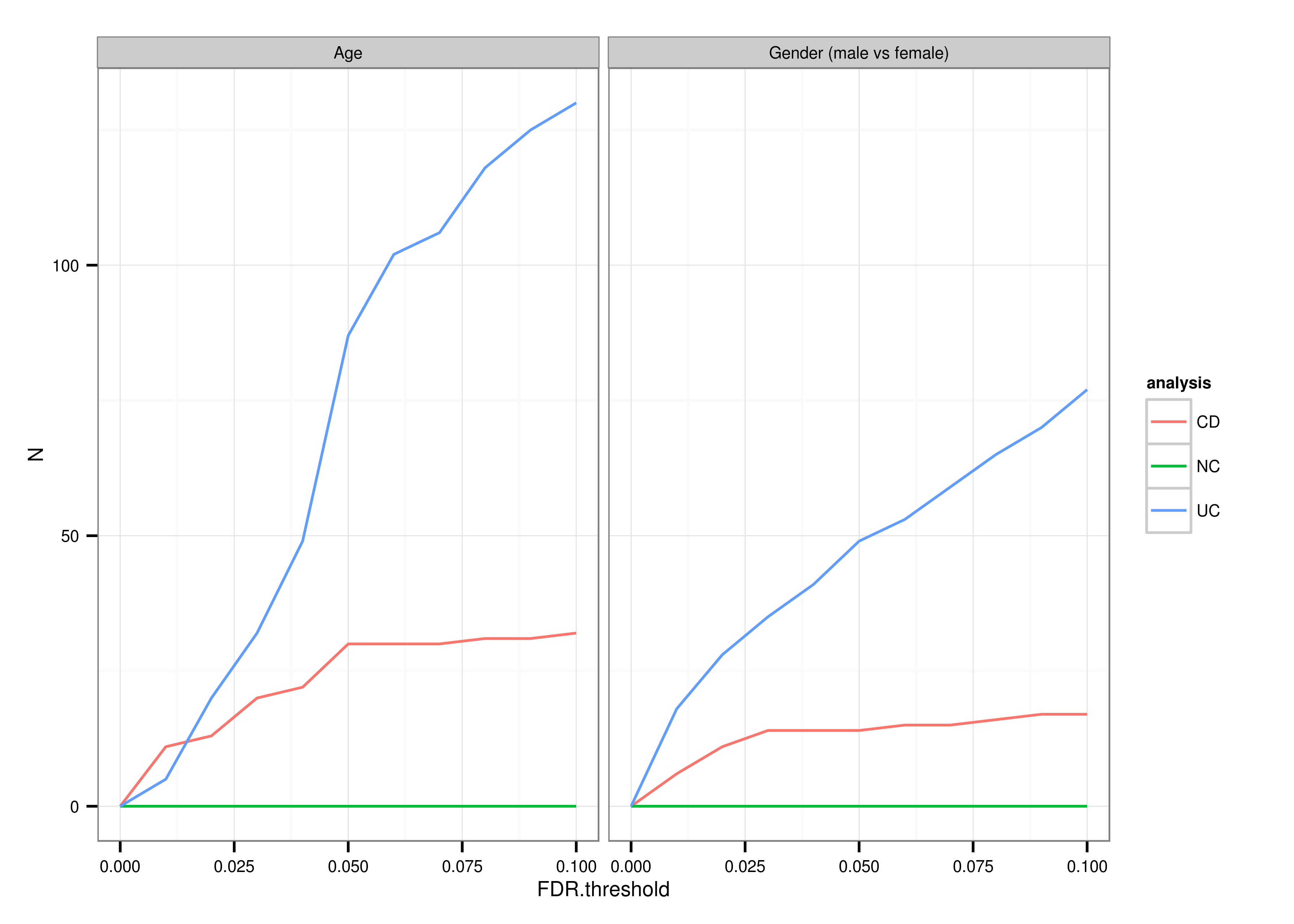

Supplement: S1 Fig — UC dominates CD and NC for both Age (left panel) and Sex (right panel). (PNG) [file pgen.1006565.s001.png]

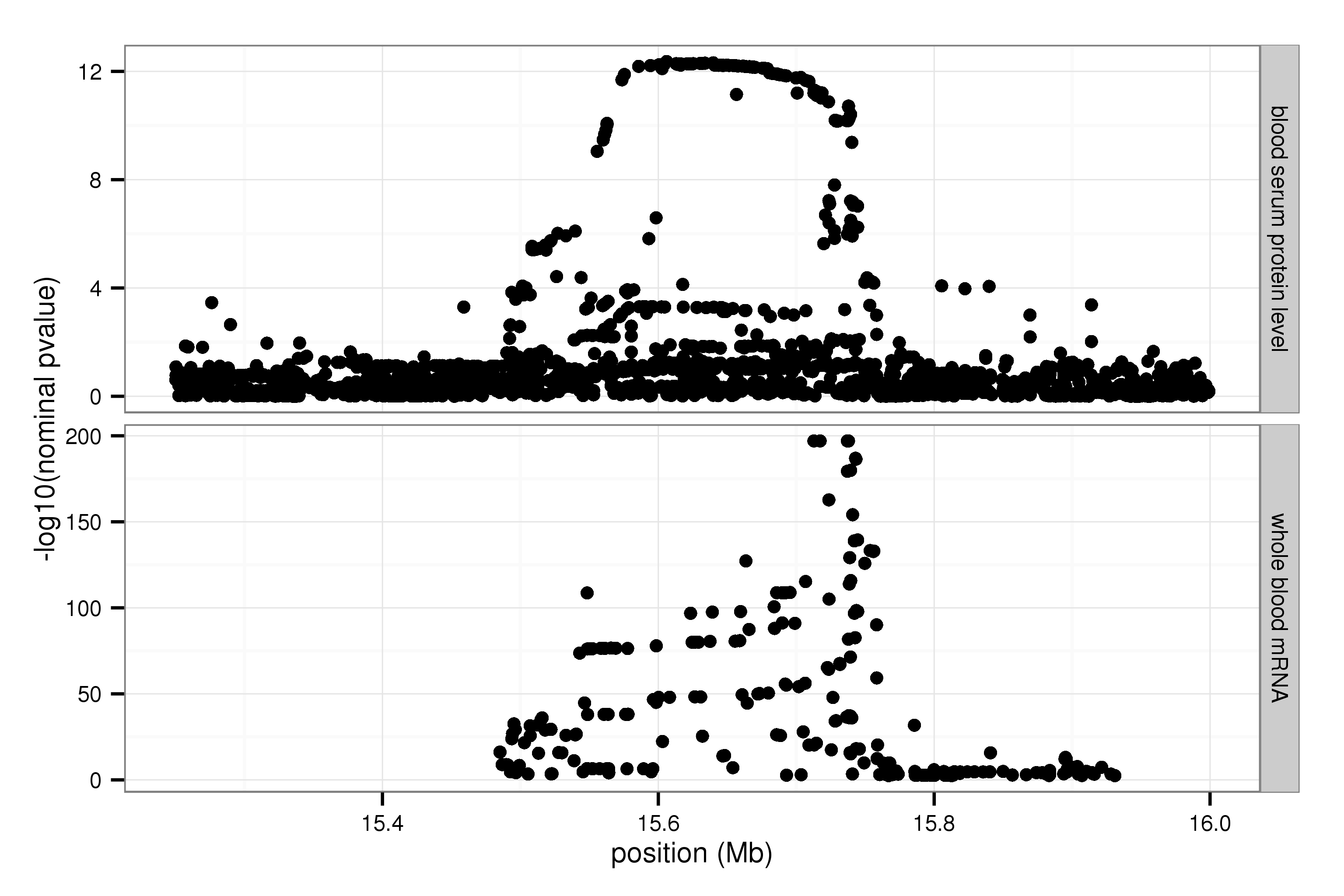

Supplement: S2 Fig — (PNG) [file pgen.1006565.s002.png]

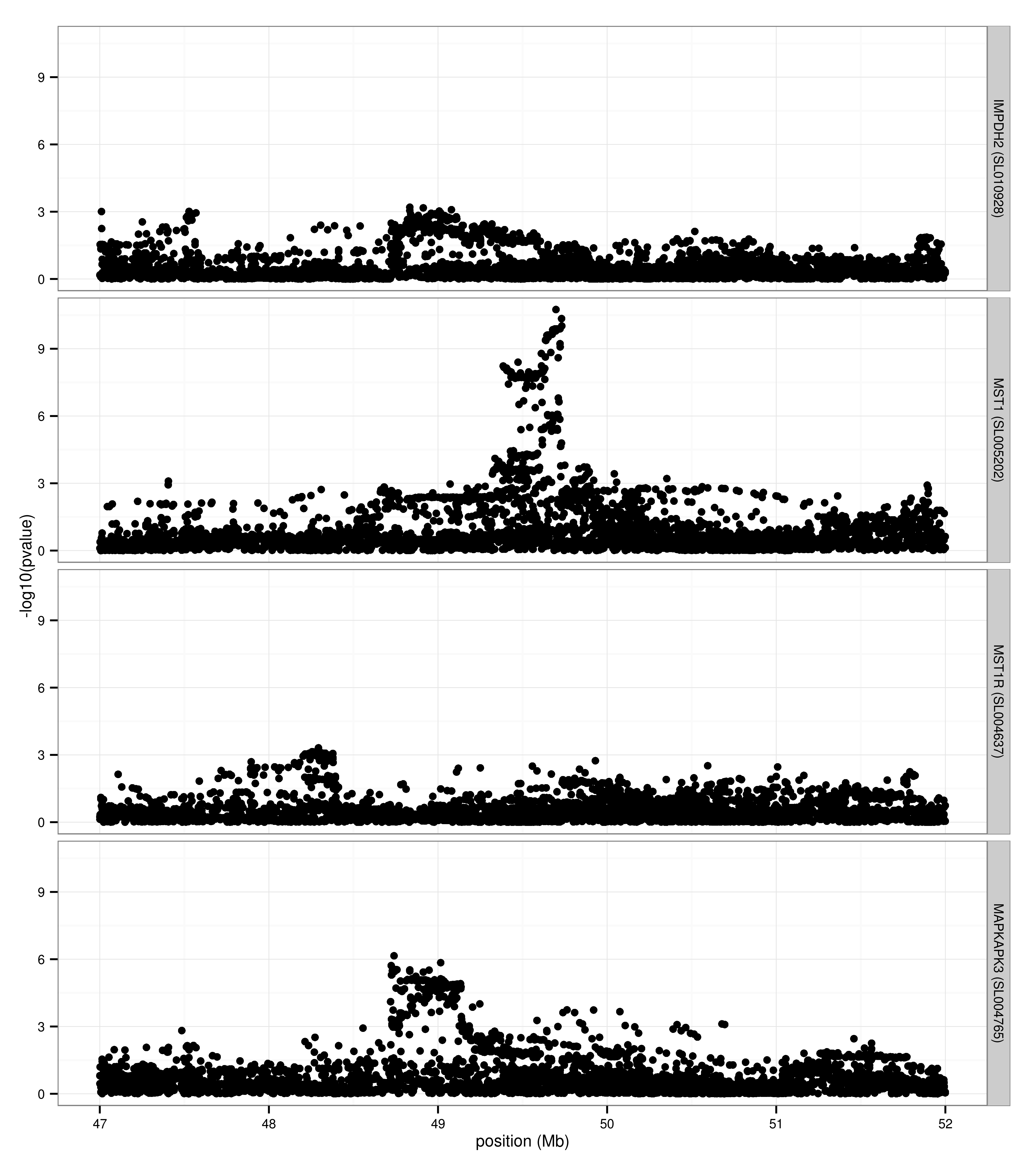

Supplement: S3 Fig — (PNG) [file pgen.1006565.s003.png]

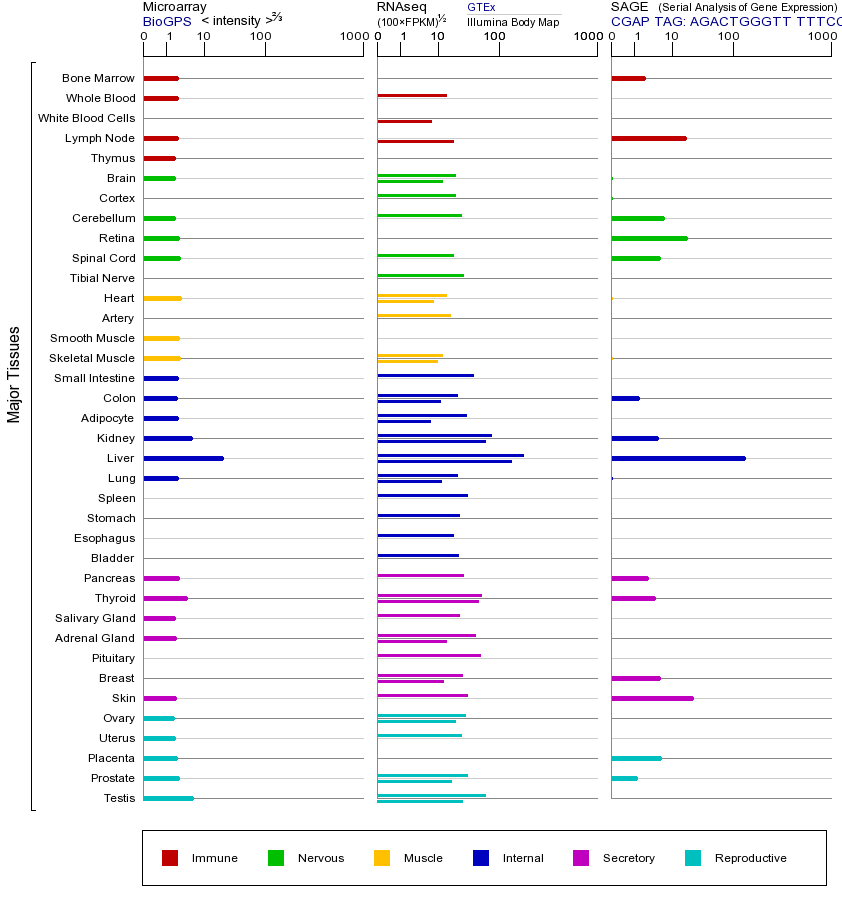

Supplement: S4 Fig — Image retrieved from GeneCards (www.genecards.org). It shows data pooled from BioGPS (biogps.org), GTex (www.gtexportal.org), and SAGE (cgap.nci.nih.gov/SAGE). (PNG) [file pgen.1006565.s004.png]

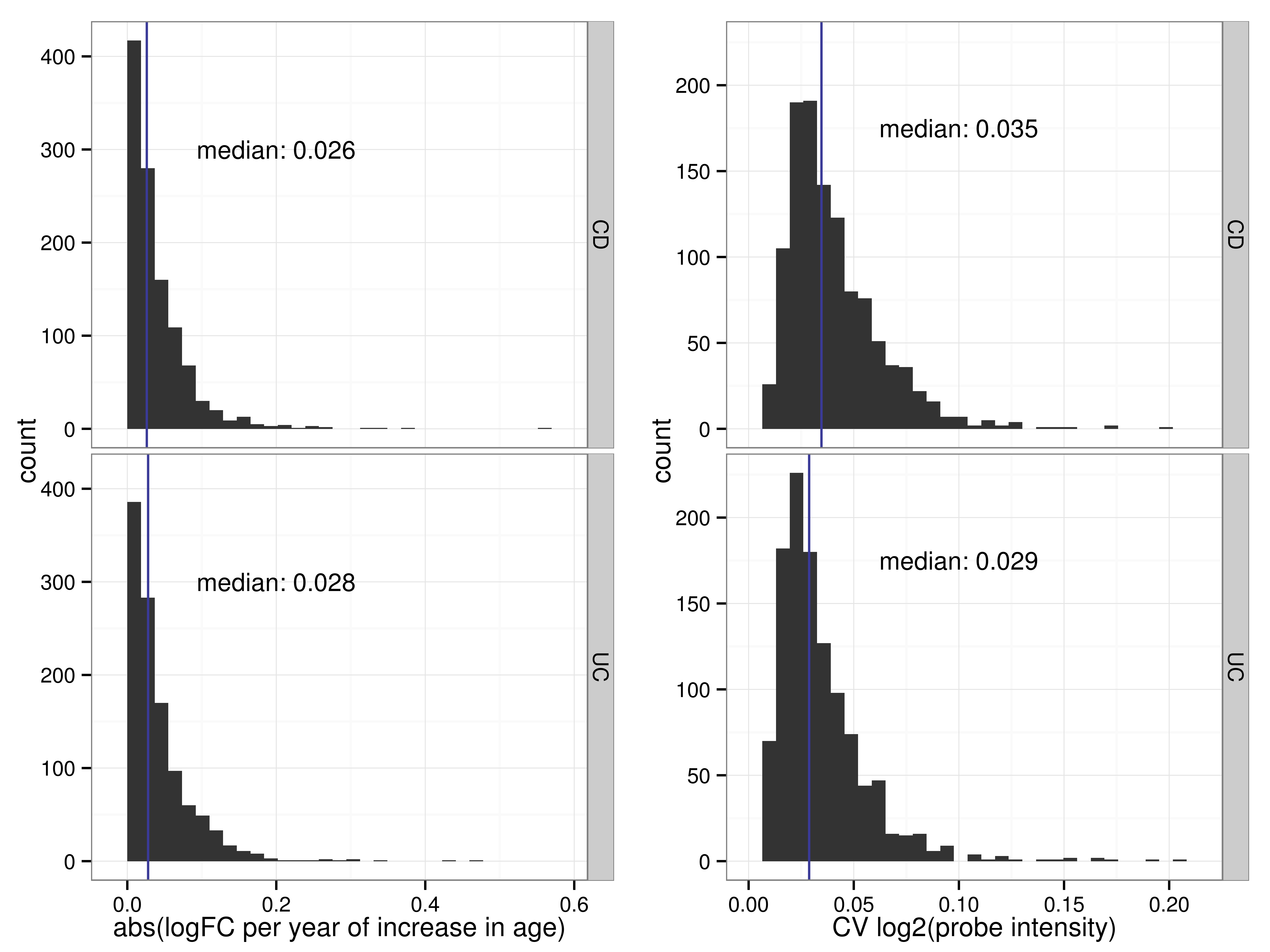

Supplement: S5 Fig — Difference in median absolute effect sizes between the two cohorts is not significant (Wilcoxon test p = 0.188). Difference in the coefficient of variation (SD/mean) is significant (Wilcoxon test p = 1.32e-14). (TIFF) [file pgen.1006565.s005.tiff]

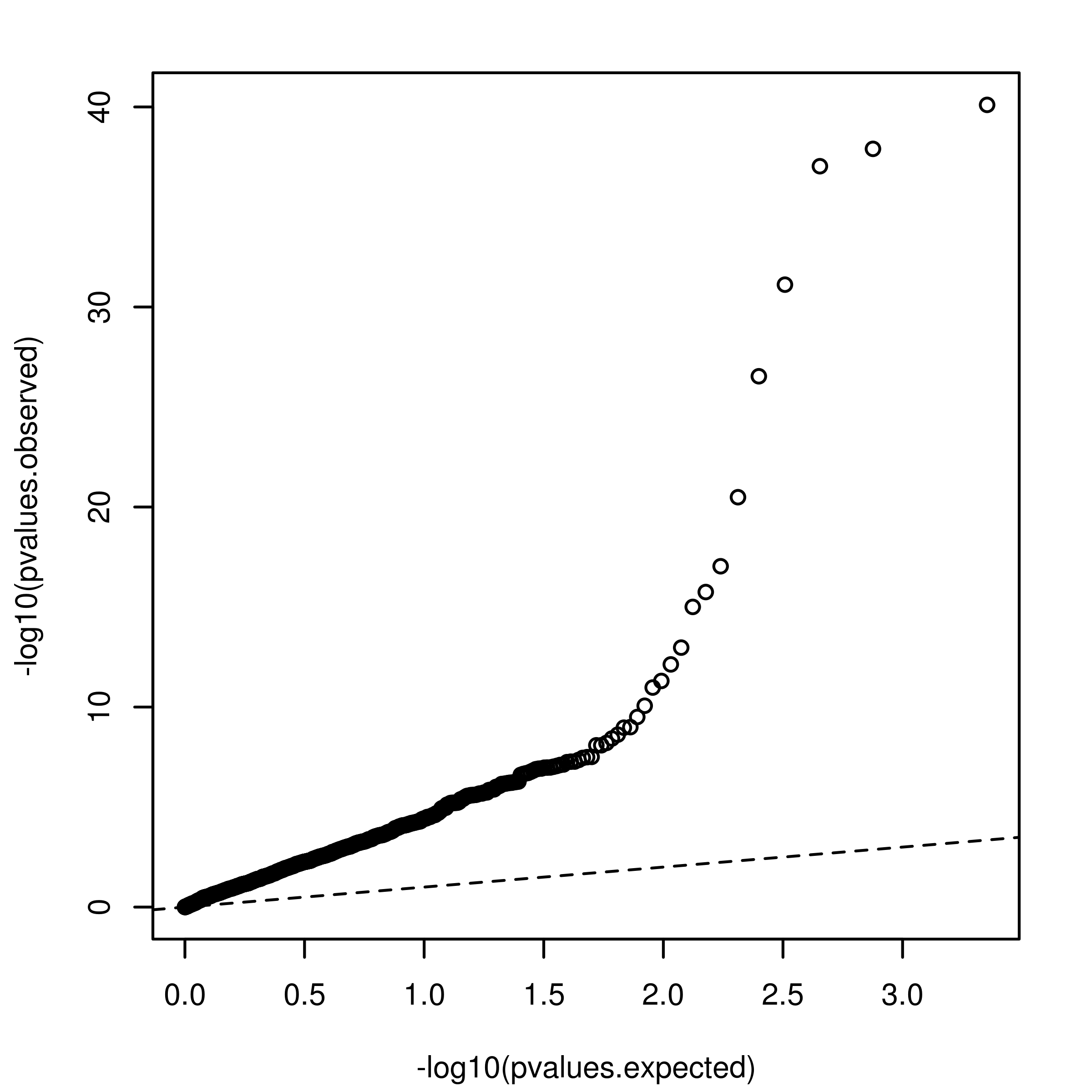

Supplement: S6 Fig — Qqplot showing -log10(pvalue)s expected under the null hypothesis of no batch effect (horizontal axis) and observed Kruskal-Wallis test pvalues of batch effect (vertical axis); each circle represents a single tested probe. For each probe, Kruskal-Wallis test was performed testing that the ‘location’ of the log-intensity of the probe was the same across the 5 available batches (271 samples, 4 degrees of freedom Kruskal-Wallis test). (TIFF) [file pgen.1006565.s006.tiff]

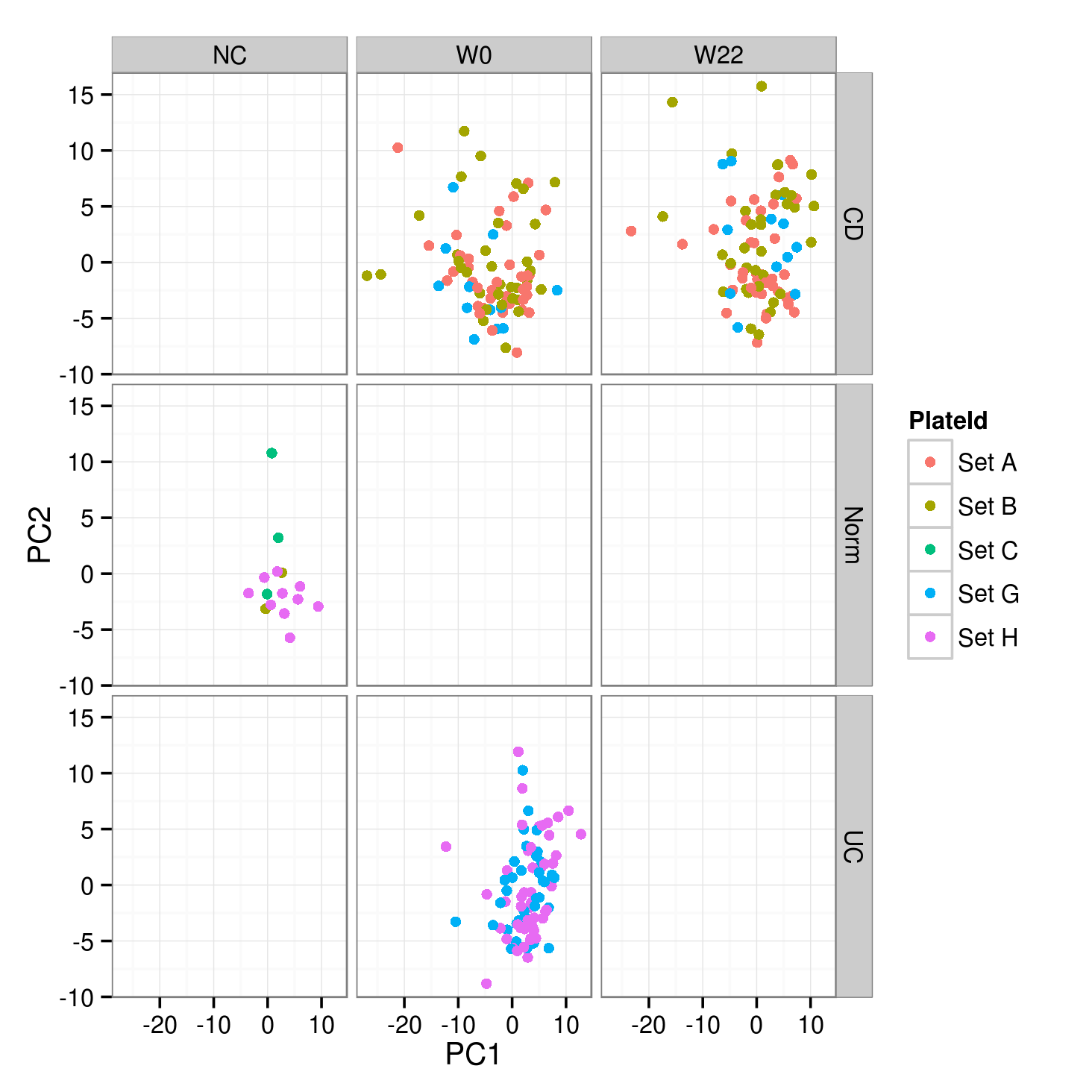

Supplement: S7 Fig — First two principal components (PC1 on the horizontal axis, PC2 on the vertical axis) with samples stratified by disease status (panel rows) and time point (panel columns) and color coded by array plate. (PNG) [file pgen.1006565.s007.png]

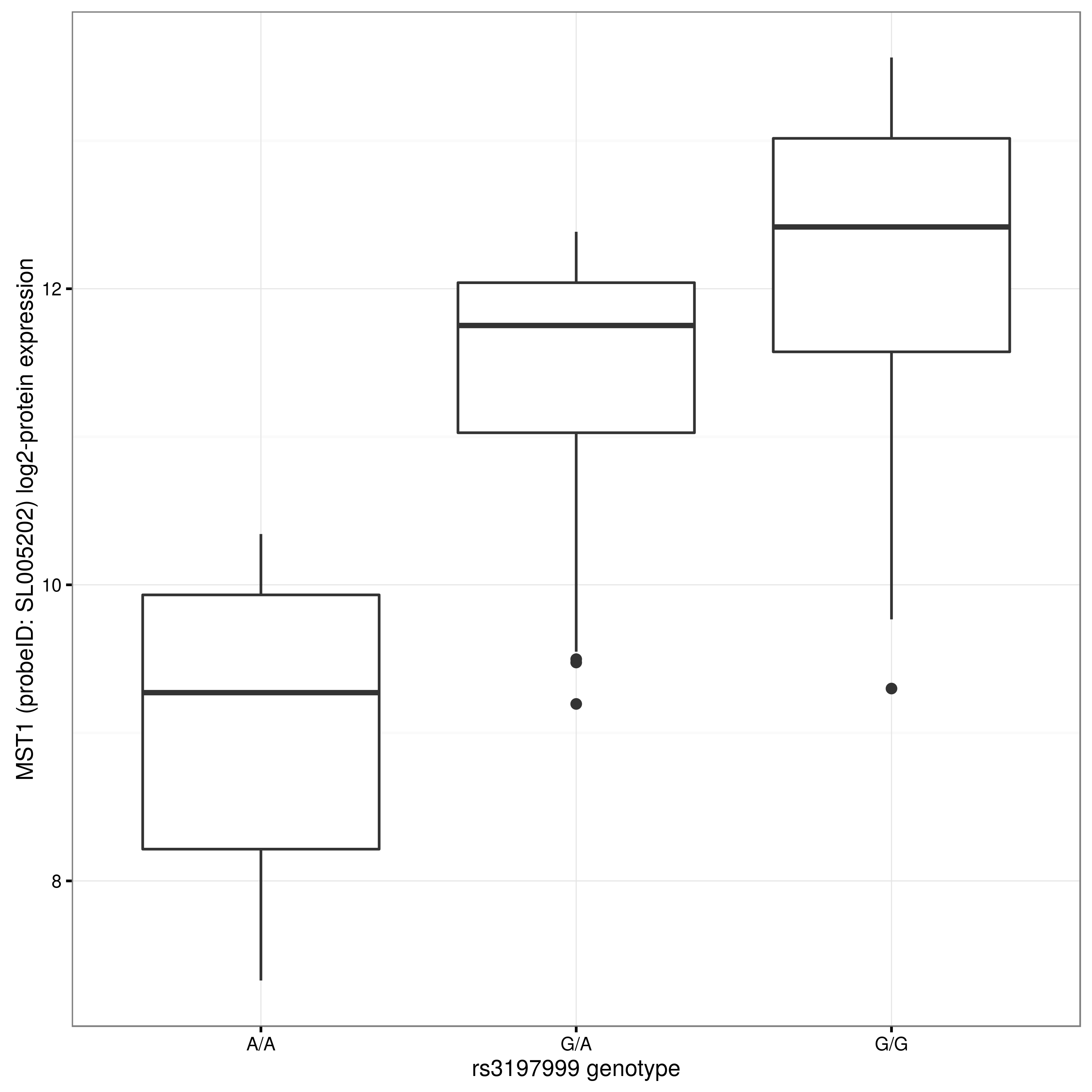

Supplement: S8 Fig — (PNG) [file pgen.1006565.s008.png]
